# Soft tissue reconstruction of the foot after mine-explosion injury: a case report

**DOI:** 10.1080/23320885.2026.2665055

**Published:** 2026-05-14

**Authors:** O.V. Pasichnyk, T.I. Tamm, I.M. Mamontov, D.V. Pryimak

**Affiliations:** Department of Surgery, Kharkiv National Medical University, Kharkiv, Ukraine

**Keywords:** Gunshot wound, mine-explosion injury, vacuum-assisted closure system (VAC), local wound therapy, foot reconstruction

## Abstract

A 22-year-old male sustained a high-energy blast injury after stepping on an anti-personnel mine during combat operations in eastern Ukraine, resulting in an extensive plantar soft tissue defect predominantly involving the central arch, while preserving critical weight-bearing structures, including the calcaneal tuberosity and metatarsal heads. The aim of this report is to describe a staged reconstructive strategy and evaluate the clinical effectiveness of prolonged negative pressure wound therapy (NPWT) combined with delayed split-thickness skin grafting for functional limb salvage in complex combat-related injuries. The patient was managed using a structured multi-stage protocol that included repeated radical debridement to achieve a viable wound bed, prolonged NPWT to promote granulation tissue formation, reduce edema, and control exudation, along with gradual wound edge approximation using retention sutures. Targeted antimicrobial therapy was administered according to microbiological findings, followed by delayed split-thickness skin grafting and a structured rehabilitation program. Complete graft integration was achieved without purulent-septic complications. At 6.5 months of follow-up, the patient demonstrated restoration of full weight-bearing capacity, preservation of plantar contour and biomechanics, stable gait, and a satisfactory functional outcome without chronic ulceration, infection, or neuropathic pain. This case highlights the clinical value of an integrated, stepwise reconstructive approach in managing severe blast-induced plantar defects and underscores the importance of prolonged NPWT as a key modality for optimizing wound bed preparation and improving reconstructive outcomes when critical anatomical structures are preserved.

## Introduction

Mine-explosion injuries (MEIs) of the lower extremities remain a major challenge in military surgery and are characterized by extensive soft tissue defects, high infection risk, blast-induced tissue destruction, and microvascular compromise [1–3]. High-energy mechanisms generate a complex zone of injury extending beyond visibly damaged tissues, often described as the ‘zone of occult injury’ or ‘molecular shock zone’, where cellular and vascular damage may progress over time [4,5]. Choosing the optimal reconstructive strategy is critical for limb salvage and functional recovery [1–3].

Reconstruction of plantar soft tissues presents particular difficulty because the specialized structure of plantar skin must be considered. The plantar surface is characterized by a thick stratum corneum, dense collagen architecture, fibrous septa firmly anchoring skin to underlying fascia, and rich innervation—features essential for shock absorption, load resistance, and sensory feedback [6]. Therefore, reconstructive strategies must aim not only at defect closure but also at restoration of functional weight-bearing properties.

Given these challenges, selecting an appropriate reconstructive algorithm is critical for limb salvage and long-term functional recovery [1,2,4,5].

## Objective

To present an effective reconstructive algorithm for combat-related foot trauma using NPWT and plastic surgical techniques according to the principles of the reconstructive ladder and reconstructive elevator.

## Case presentation

**Patient**: Male, 22 years old.

The patient sustained a high-energy blast injury after stepping on an anti-personnel mine during active combat operations in eastern Ukraine.

Initial surgical management was performed by a Forward Surgical Team (Role 2), including primary debridement and hemorrhage control. The patient was stabilized and transferred to a Role 3 facility for definitive care. A tourniquet was applied immediately after injury and remained in place for 1 h. Evacuation time to Role 2 was approximately 1 h, and to Role 3 approximately 3 h. Despite difficult evacuation conditions, surgical management occurred within an extended “golden hour” framework, minimizing secondary ischemic damage.

On admission, the diagnosis was: blast injury with comminuted fractures of the 1st-5th metatarsals of the left foot; multiple fragmentary gunshot wounds to extremities; extensive soft tissue defect of the left foot.

**Figure 1.**
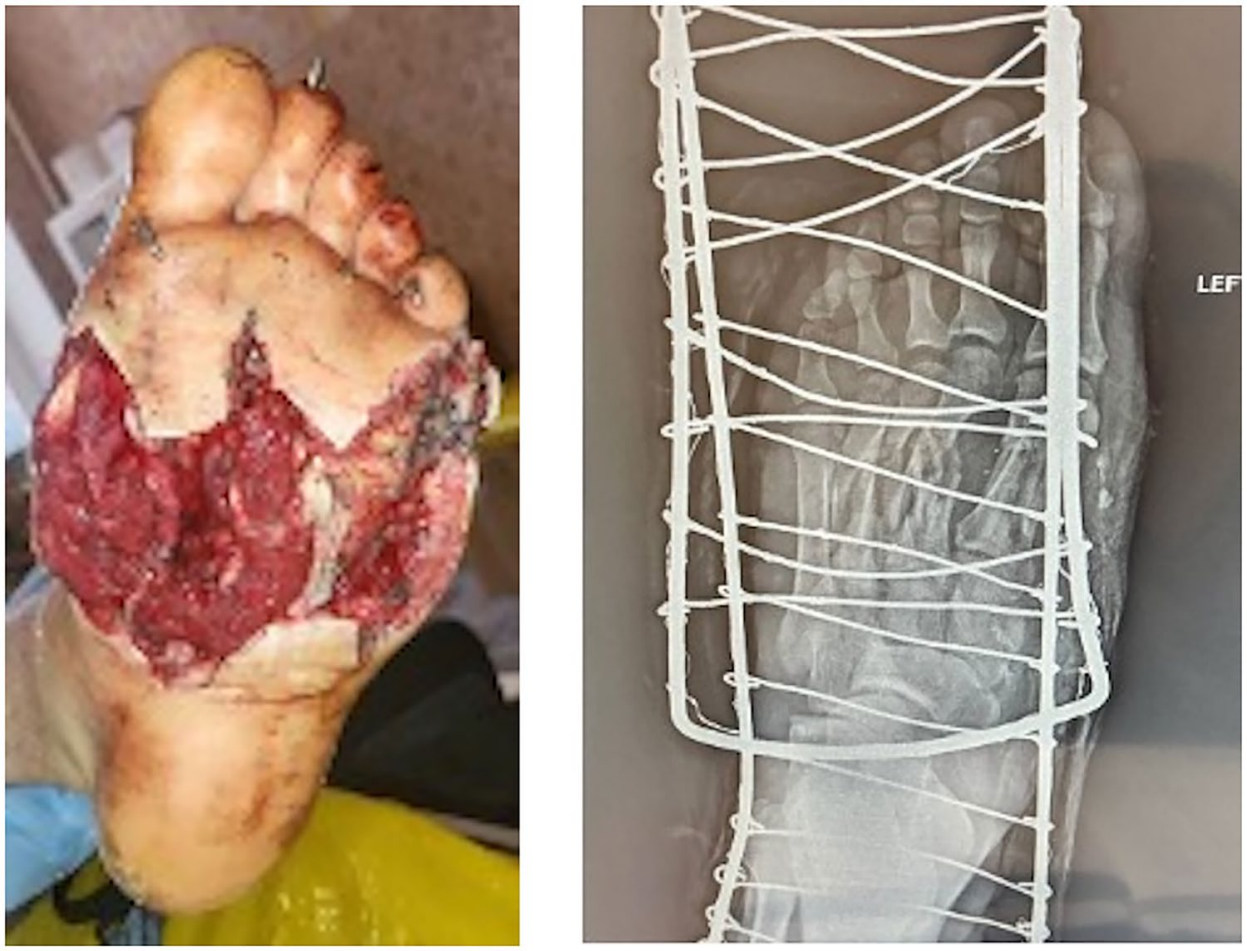
A - Clinical photograph on admission showing irregular 11x8 cm plantar defect with necrotic edges, exposed tendons, hyperemia and contamination; B - Plain X-ray demonstrating comminuted fractures of 1st–5th metatarsals stabilized with Kirschner wires, no major foreign bodies.

**Locus morbi:** Marked swelling of the left foot. On the plantar surface, an irregular wound measuring approximately 11 × 8 cm with a deep soft tissue defect extending to subcutaneous fat and muscle, centrally exposed tendons; necrotic wound edges; surrounding hyperemia and contamination. On the dorsal surface, two fasciotomy wounds (∼8 × 2 cm and ∼7 × 1.5 cm). Kirschner wires were visible in the region of the 1st–5th metatarsals. Distal sensitivity was decreased (Figure 1(A,B)).

The defect predominantly involved the central plantar arch while sparing the calcaneal tuberosity and metatarsal heads, which later proved critical for successful reconstruction.

Additional blind wounds up to 2 cm were present in the proximal third of the right shoulder; multiple tangential wounds were observed on the right forearm, thigh, and lower leg. No life-threatening thoracoabdominal or cranial injuries were identified.

Perfusion was assessed clinically (capillary refill <2 s, warm skin, adequate bleeding from wound edges) and confirmed by handheld Doppler ultrasound, demonstrating preserved pulsations of the dorsalis pedis and posterior tibial arteries. Formal angiography was not required due to stable clinical perfusion.

Microbiological examination: Staphylococcus aureus at 10^6^ CFU/mL indicating high bacterial contamination. Empiric antibiotic therapy with Ceftractam (cefoperazone + sulbactam) was initiated, active against gram-positive and gram-negative organisms including beta-lactamase-producing S. aureus. Final adjustments were planned post antibiogram.

Initial management followed damage control surgery principles with radical debridement and delayed definitive closure.

## Treatment course

**Figure 2.**
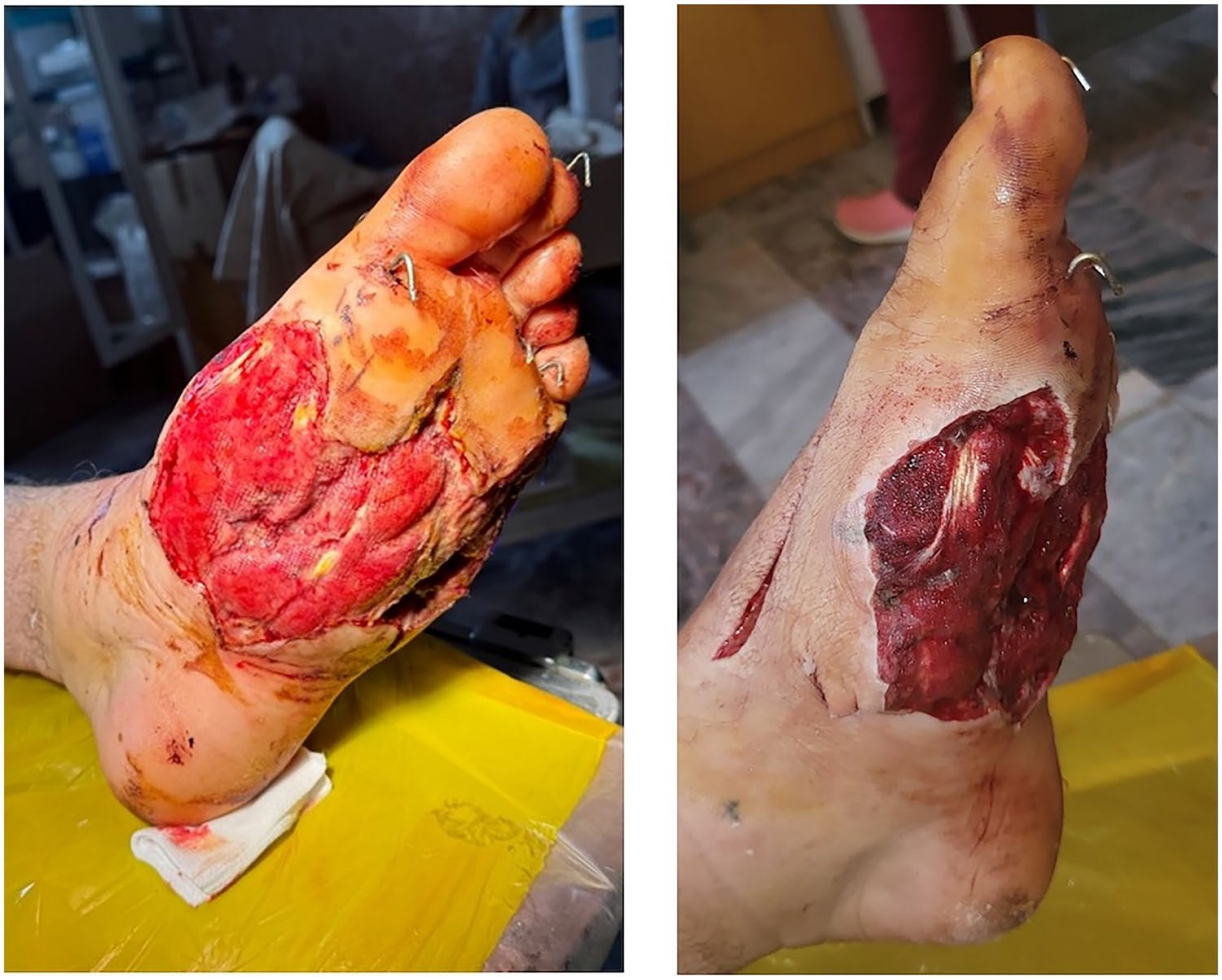
Day 7 of first VAC cycle: healthy granulation tissue covering the wound bed, visisble wound contraction under Prolene retention sutures (defect reduced by ∼30%).

**Day 3**: Secondary radical debridement with maximal preservation of viable skin margins. NPWT was applied for 7 days. Retention sutures (Prolene 1/0) were placed over the VAC dressing to achieve progressive wound edge approximation (as shown in Figure 2). This approach corresponds with traction-assisted NPWT techniques described in the literature [7,8].

**Figure 3.**
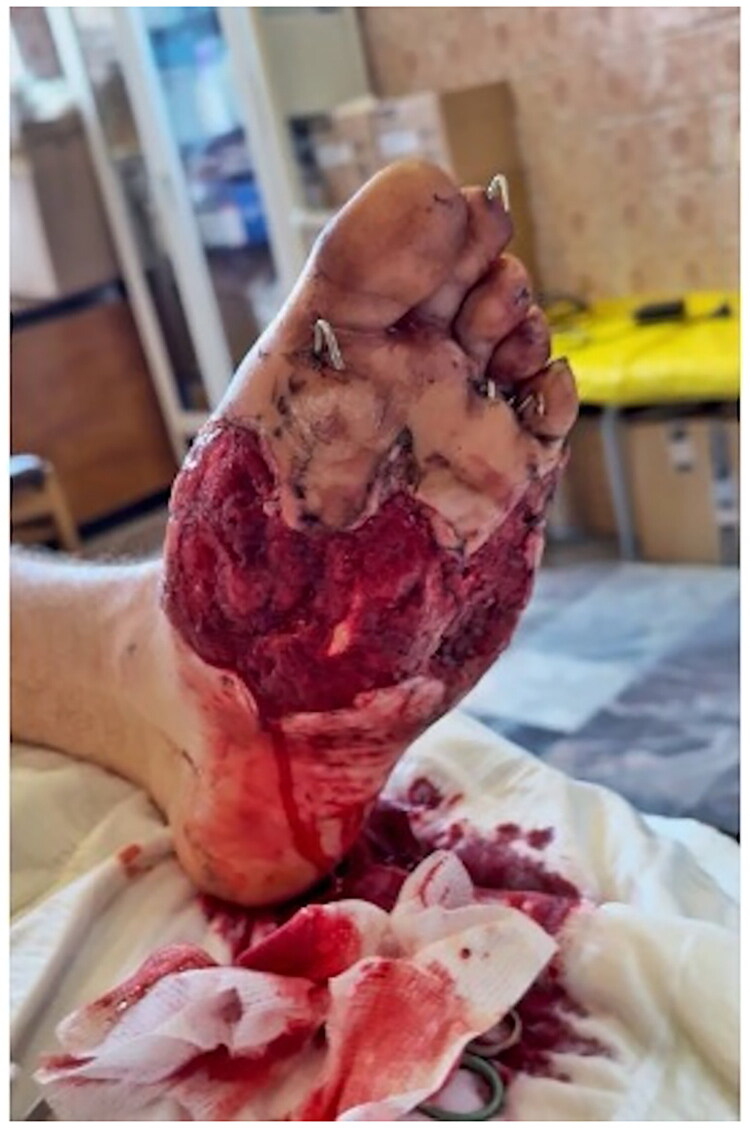
Day 10, second VAC cycle: further improvement with robust granulations, decreased edema and progressive edge approximation.

**Day 10**: Repeat debridement and second 7-day NPWT cycle (Figure 3).

**Days 7–22**: Local treatment with Pyophage (bacteriophage solution) and Arederma (antiseptic spray). A short tapering course of dexamethasone (24-16-8 mg) was administered to reduce severe inflammatory edema [9].

**Figure 4.**
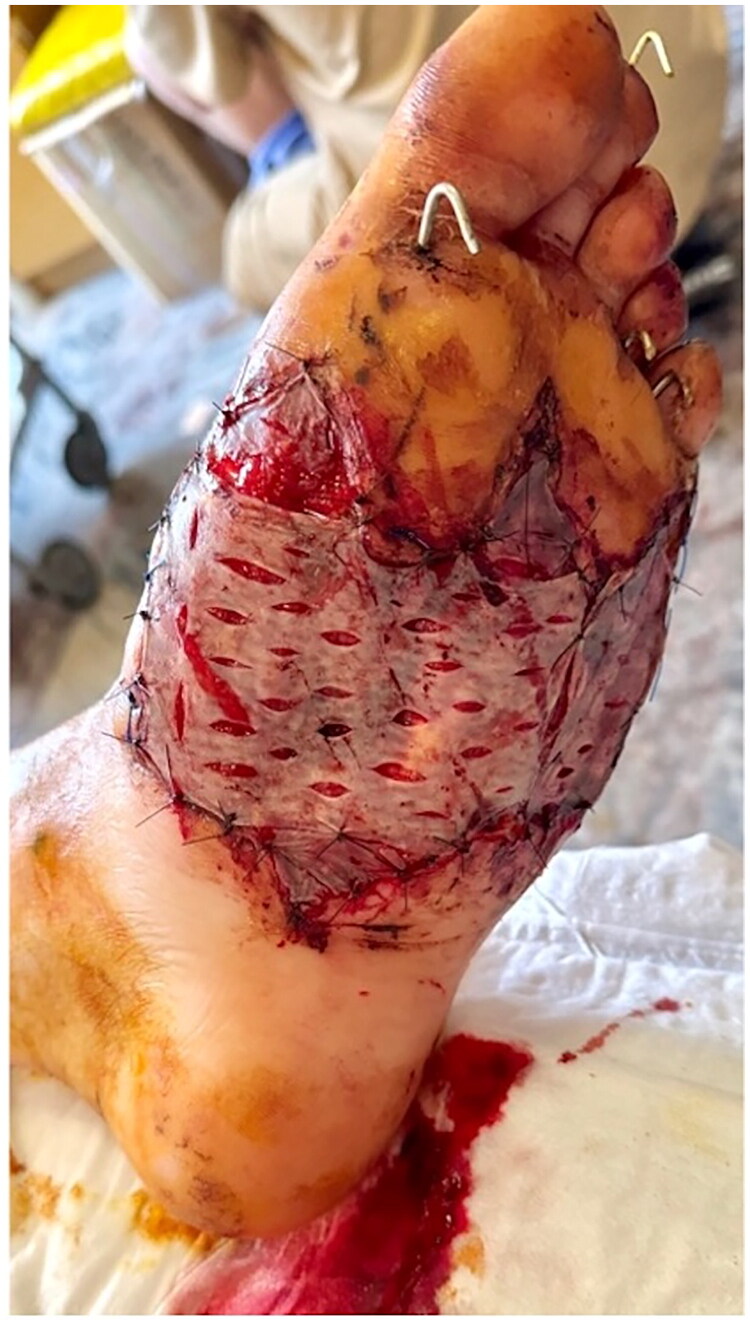
Day 22: split-thickness skin graft placement – perforated, secured with sutures, good adherence to prepared bed.

**Day 22**: Split-thickness skin grafting (7 × 13 cm, 0.4 mm thickness). The graft was perforated and secured with sutures (Figure 4).

**Figure 5.**
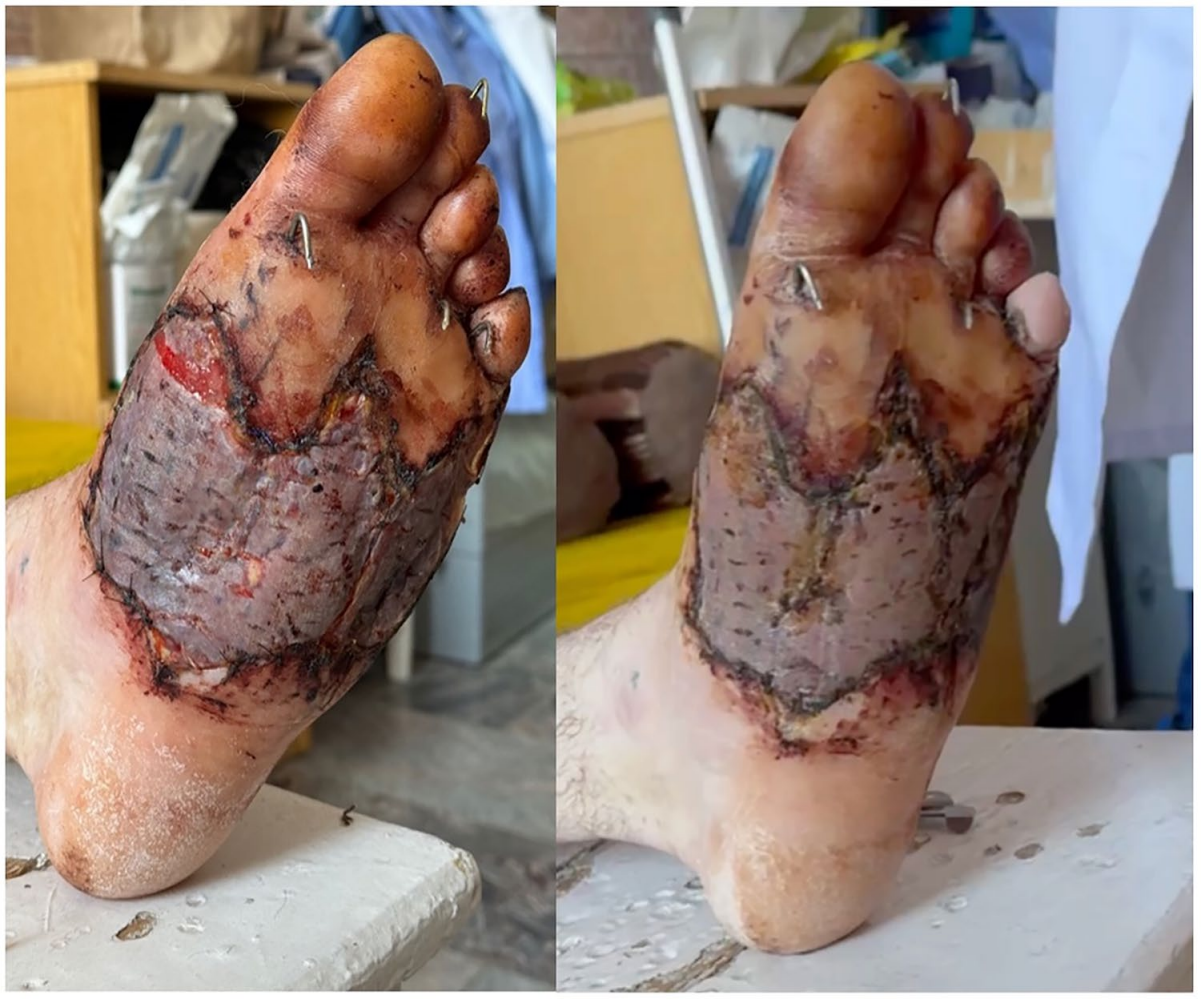
Graft maturation: A - 10 days post-grafting, early re-epitalization; B −23 days, near-complete take with stable coverage.

**Postoperative care**: Postoperative management included antibiotic therapy, physiotherapy, and graft monitoring (Figure 5(A,B))

**Rehabilitation included:**
- Individualized physical therapy for joint mobilization
- Supervised kinesiotherapy
- Manual lymphatic and microcirculatory support
- Gradual protected weight-bearing in orthopedic footwear
- Progressive load adaptation monitoring

## Outcomes (6.5 months post-injury)

**Figure 6.**
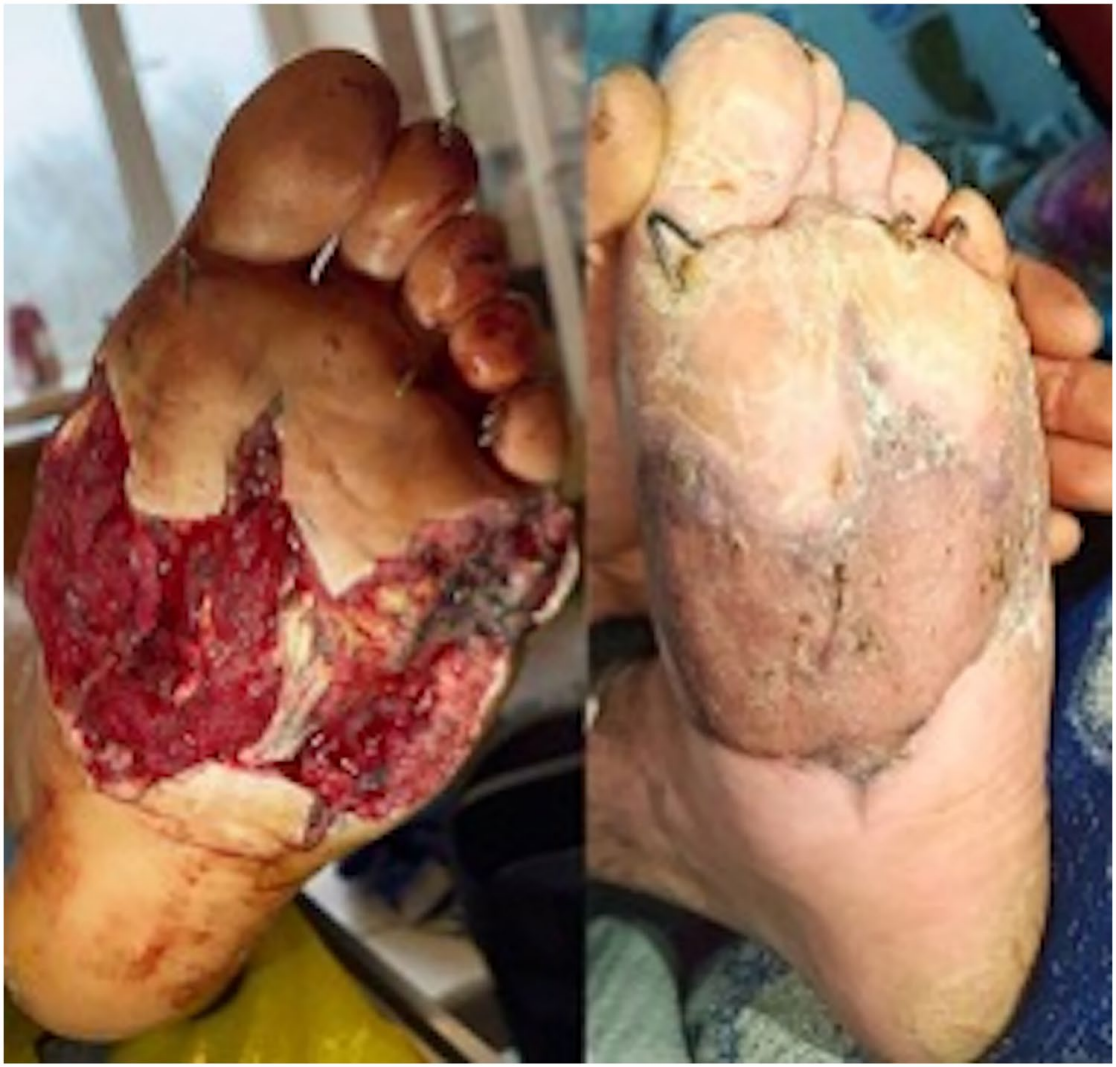
Final result at 6.5 months: full graft integration, preserved plantar contour, restored weight-bearing capacity without contractures, ulcers or trophic changes.

At 6.5 months, the patient achieved full weight-bearing ambulation in orthopedic footwear without chronic pain, ulceration (Figure 6). Plantar contour was preserved, and no trophic complications were observed.

## Discussion

The management strategy in this case aligns with contemporary Ukrainian and international experience in treating mine-explosion injuries and reflects current advances in understanding early tissue damage and prognostic assessment [1,5,10,11,12]. Blast trauma differs significantly from conventional ballistic wounds due to the extensive zone of occult injury characterized by progressive microvascular compromise and delayed necrosis [4]. Therefore, serial staged debridement is essential.

NPWT plays a central role in modern combat wound management. Evidence from Cochrane analyses and trauma studies confirms its effectiveness in reducing infection risk, promoting granulation tissue formation, and facilitating staged closure [13]. The use of retention sutures in combination with NPWT has been described as a method to gradually reduce wound size and tension, potentially avoiding more complex flap reconstruction [7,8,14].

Similar challenges in foot reconstruction after landmine injuries have been reported in Ukrainian and other modern conflicts (Iraq, Afghanistan), where high-energy blast mechanisms cause extensive soft tissue and bone damage, often leading to higher amputation rates and complex reconstructive needs. These studies reinforce the value of staged NPWT and skin grafting over complex flaps in contaminated, resource-limited settings, particularly when high-load-bearing zones are spared [10,11].

Recent Ukrainian wartime surgical experience has further expanded the understanding of staged management of mine-explosive extremity injuries. Structured serial debridement combined with controlled NPWT has been shown to improve wound bed preparation in contaminated blast injuries and reduce secondary necrosis by addressing the extended zone of occult microvascular damage [12,15]. These findings emphasize that in high-energy mine trauma, tissue viability cannot be reliably determined during the first surgical intervention due to the evolving molecular shock zone.

Lurin et al. described the implementation of protocol-based VAC therapy in combat support hospitals during the war in Ukraine, highlighting pressure modulation strategies (−75 to −125 mmHg depending on tissue viability and exudate volume) and the importance of delayed definitive closure after repeated wound reassessment [16]. Their data demonstrate improved limb salvage rates even under resource-constrained battlefield conditions.

In addition, Ukrainian military surgeons have reported the adjunctive use of digital infrared thermography as a non-invasive tool for real-time perfusion assessment in blast and gunshot injuries, allowing earlier detection of marginal ischemia and better timing of reconstructive stages [17]. Although thermography was not used in the present case, its integration into standardized battlefield algorithms may further optimize reconstructive decision-making.

These contemporary wartime data confirm that prolonged NPWT combined with staged reconstruction is not merely a supportive measure but a central element of modern limb salvage strategy in blast-related foot injuries.

In contrast to complex flap reconstruction, split-thickness skin grafting was possible due to preservation of the calcaneal tuberosity and metatarsal heads. Sparing high-load-bearing zones is a decisive factor in selecting reconstructive options [6,18,19].

Rehabilitation following combat trauma is equally critical. WHO guidelines emphasize early multidisciplinary rehabilitation in emergency medical teams to prevent long-term disability [20]. Ukrainian rehabilitation data further support structured physical therapy following blast injuries [21].

Thus, this case contributes additional clinical evidence supporting staged NPWT-based reconstruction for plantar blast defects when anatomical conditions permit.

## Conclusion

The presented clinical case demonstrates the feasibility of limb preservation even after severe mine-explosion injury of the foot. The combined use of staged radical debridement, prolonged NPWT with retention sutures, delayed split-thickness skin grafting, and structured rehabilitation resulted in successful functional recovery without purulent-septic complications. An individualized, multi-stage reconstructive strategy remains essential in managing complex combat-related plantar injuries.

### Limitations

Single case report; absence of standardized quality-of-life assessment (e.g. SF-36); no formal gait laboratory analysis performed.
